# Supplementary material for: Human Saposin B Ligand Binding and Presentation to α-Galactosidase A
Source: bioRxiv. 2024 Apr 4:2024.04.04.584535. Preprint. [Version 1] doi: 10.1101/2024.04.04.584535 (PMC11014568; doi:10.1101/2024.04.04.584535)
Supplement: 1 [file NIHPP2024.04.04.584535v1-supplement-1.pdf]

bioRxiv preprint doi: https://doi.org/10.1101/2024.04.04.584535; this version posted April 4, 2024. The copyright holder for this preprint (which was not certified by peer review) is the author/funder, who has granted bioRxiv a license to display the preprint in perpetuity. It is made available under aCC-BY 4.0 International license.

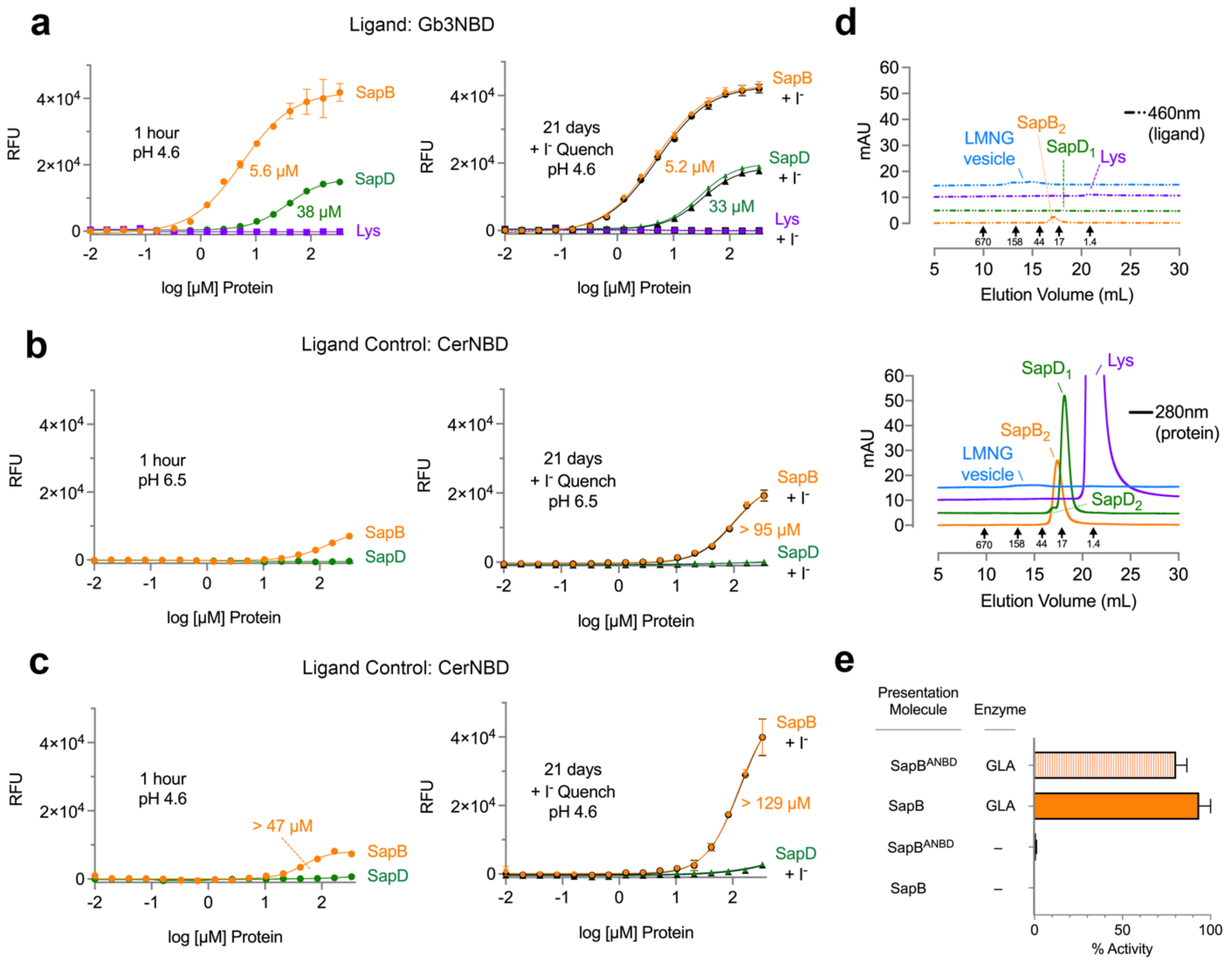

Supplementary Figure 1 | Specificity and ligand controls for SapB binding to Gb3-NBD. a The equilibrium binding of SapB (orange circle), the saposin specificity control SapD (green triangle), and negative control lysozyme (Lys, purple square) to Gb3-NBD after one hour (*left*) or after twenty-one days with iodide quenching (*right*) at pH 4.6. The apparent dissociation constants are labeled. **b** SapB and SapD binding to the ligand control Cer-NBD, which lacks the glycan of Gb3, after one hour (*left*) or twenty-one days later with iodide quenching (*right*) at pH 6.5. **c** The same as panel b but at pH 4.6. **d** The size exclusion chromatograms of SapB (orange), SapD (green), lysozyme (purple), and LMNG (blue) without Gb3-NBD ligand, in which dual wavelength monitoring tracks both Gb3-NBD ligand (top) and protein (bottom) absorbance. **e** Hydrolysis of Gb3-NBD, measured by an increase in the concentration of free galactose, using the fluorescent reporter $SapB^{ANBD}$ (striped) or wild type SapB (orange) with and without wild type GLA. The concentration of free galactose detected was normalized to the highest value obtained by wild type SapB to show the comparative percent activity. The error bars in panels represent the standard deviation between replicates.

bioRxiv preprint doi: https://doi.org/10.1101/2024.04.04.584535; this version posted April 4, 2024. The copyright holder for this preprint (which was not certified by peer review) is the author/funder, who has granted bioRxiv a license to display the preprint in perpetuity. It is made available under aCC-BY 4.0 International license.

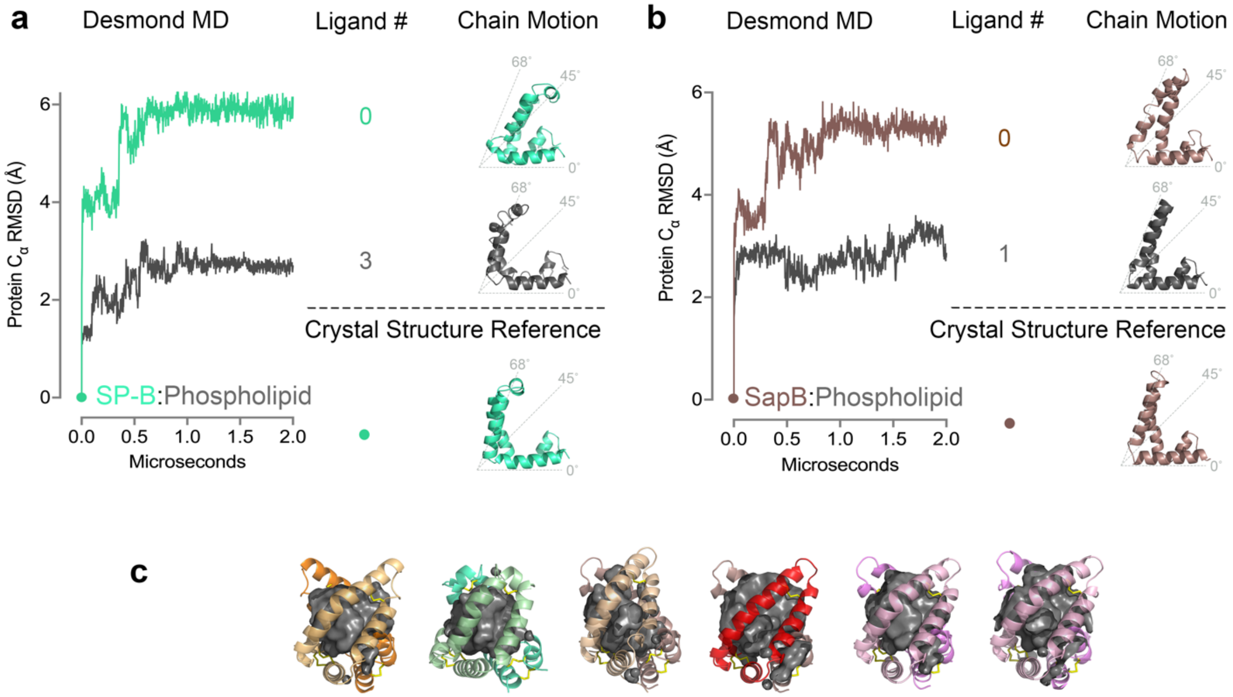

**Protein**	SapB	SP-B	SapB			
**PDB ID**	9AXG	6VYN	1N69		4V2O	
**Pocket (Å³)**	2,869	2,529	1,528	2,871	2,989	2,948
**Ligand**	Gb3NBD	Phospholipid			Chloroquine	
Built	0	3	1	0	1	2
Expected	≥ 2	3	≥ 1	≥ 2	≥ 2	≥ 2

Supplementary Figure 3 | Molecular dynamics controls for interpreting the SapB:Gb3-NBD structure as ligand-bound. a Surfactant protein B (SP-B) bound to phospholipid (green-cyan) and **b** SapB bound to phospholipid (brown) dimers were used as positive controls for the MD simulations. The alpha carbon RMSD of the protein main chains is graphed by microsecond after the simulation. Representative intrachain motions as a function of ligand presence are shown to the right. **c** A comparative analysis of the ligand-bound states of the SapB:Gb3-NBD structure and controls, including the 'unliganded' dimer of SapB bound to phospholipid (red) and the dimers of SapB bound to chloroquine (pink). The calculated pocket volumes of the saposin and saposin-like dimers are shown for comparison. The number of cargos built within the dimers of the PDB structures is indicated and an estimate of expected ligand occupancy based on the pocket volume is provided, considering SP-B as a positive control.

bioRxiv preprint doi: https://doi.org/10.1101/2024.04.04.584535; this version posted April 4, 2024. The copyright holder for this preprint (which was not certified by peer review) is the author/funder, who has granted bioRxiv a license to display the preprint in perpetuity. It is made available under aCC-BY 4.0 International license.

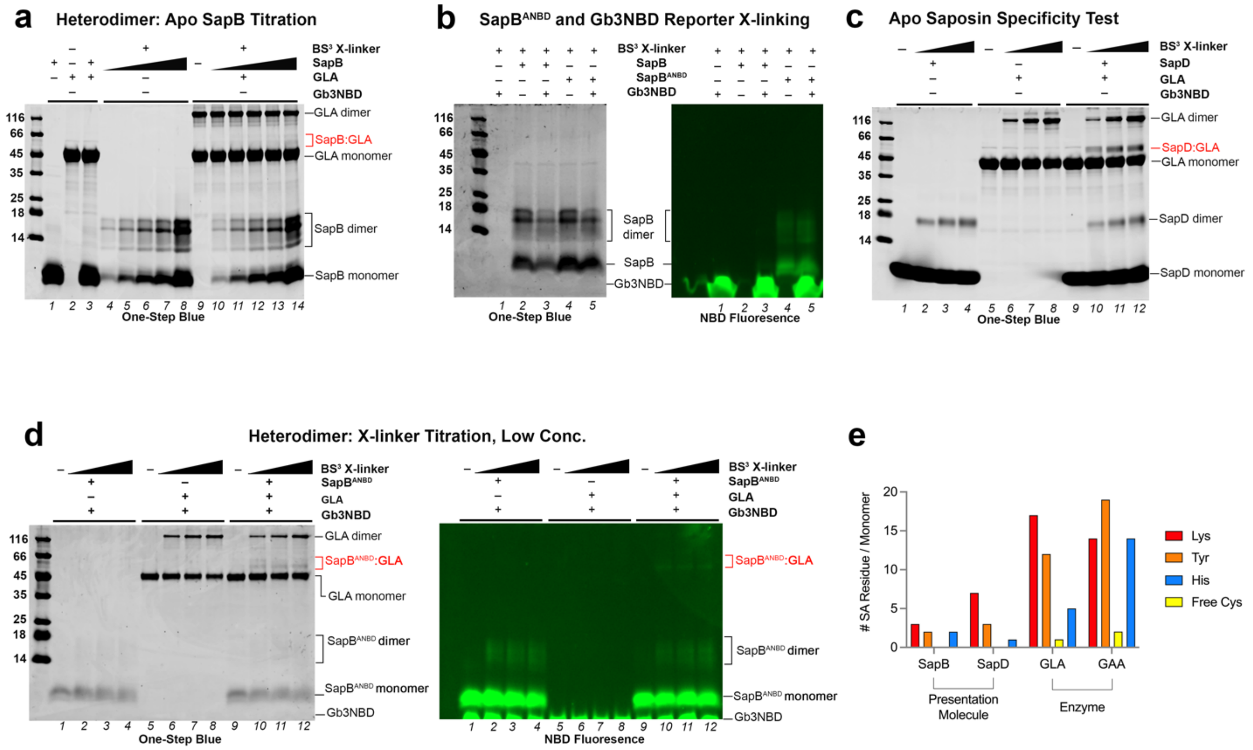

Supplementary Figure 4 | *In vitro* cross-linking controls to understand the specificity of the SapB:GLA interaction.
a One-Step Blue stained SDS-PAGE gel of 10 µM GLA and increasing concentrations (0, 25, 50, 75, 100, and 200 µM) of SapB after incubation with 1 mM BS^3 X-linker absent Gb3-NBD. **b** *Left*: SDS-PAGE of 100 µM SapB or $SapB^{ANBD}$ after incubation with 1 mM BS^3 X-linker in the presence and absence of 100 µM Gb3-NBD. *Right*: The NBD fluorescence of the SDS-PAGE gel prior to One-Step Blue staining to detect the NBD-containing bands using an XcitaBlue conversion screen and GelGreen detection filter**.** **c** SDS-PAGE gel of 10 µM GLA and 100 µM SapD samples after incubation with increasing concentrations of BS^3 X-linker (0, 0.2, 0.5, 1mM) absent Gb3-NBD. **d** *Left*: SDS-PAGE of 1 µM GLA, 10 µM $SapB^{ANBD}$, and 50 µM Gb3-NBD samples after incubation with increasing concentrations (0, 0.2, 0.5, 1 mM) of BS3 X-linker. *Right*: The NBD fluorescence of the SDS-PAGE gel prior to One-Step Blue staining. **e** A comparison graph of the number of BS^3 X-linker reactive, solvent accessible (SA) side chains (Lys-red, Tyr-orange, His-blue, free Cys-yellow) on each protein monomer from PDB structures.

bioRxiv preprint doi: https://doi.org/10.1101/2024.04.04.584535; this version posted April 4, 2024. The copyright holder for this preprint (which was not certified by peer review) is the author/funder, who has granted bioRxiv a license to display the preprint in perpetuity. It is made available under aCC-BY 4.0 International license.

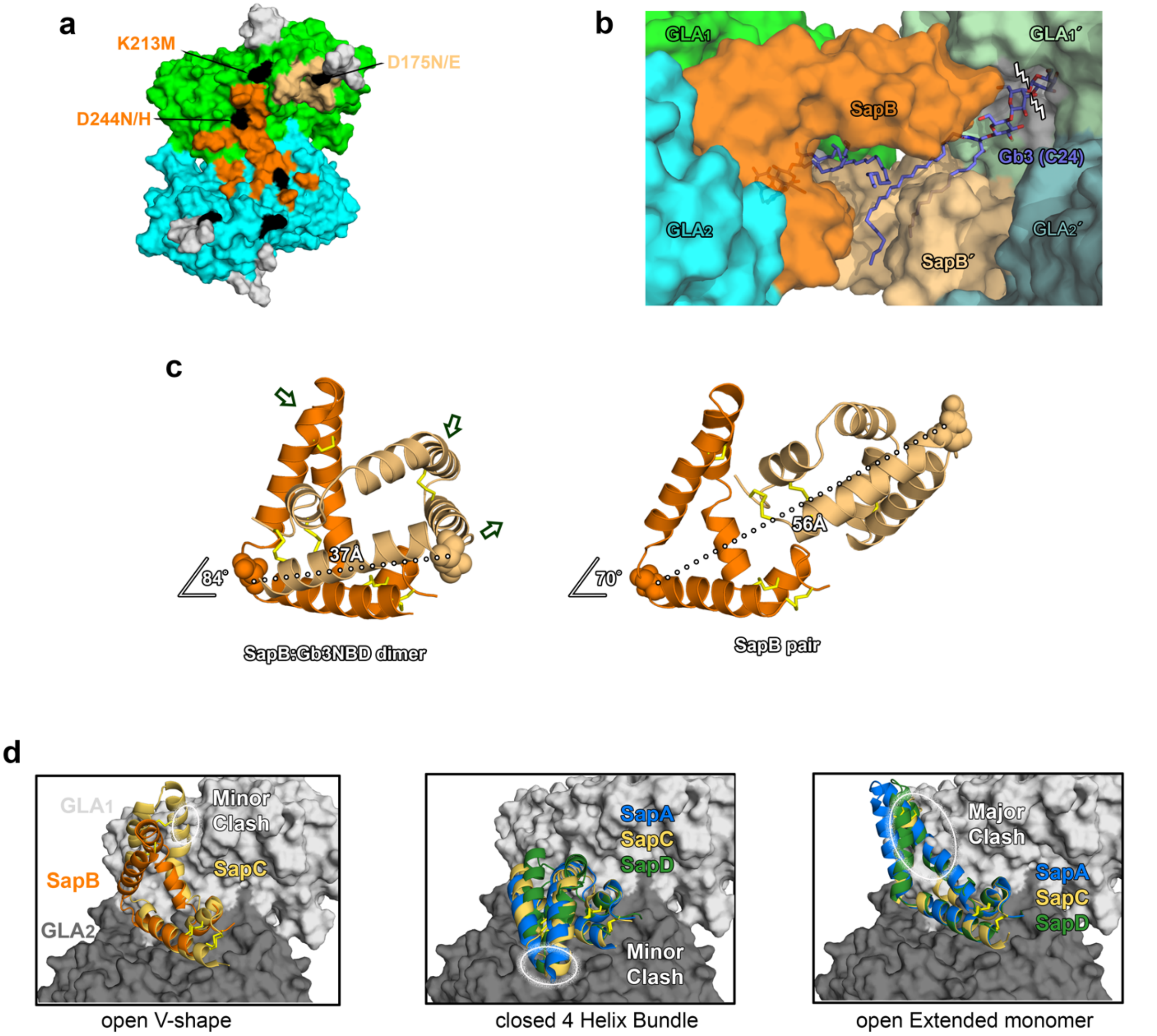

Supplementary Figure 5 | Models for the catabolism of Gb3 ligand by SapB:GLA and conformational importance. **a** Surface of the GLA dimer (green & cyan) with Fabry disease causing mutations (black) painted onto the SapB contact regions (orange & tan). Glycans are colored grey. **b** Surface of the SapB:GLA interaction with two Gb3 (C24) substrate molecules (purple sticks), manually docked according to torsion angle constraints. The structure of GLA dimer bound to melibiose was used to place the terminal αGal moiety in the $GLA_{1'}$ (pale green) active site (grey) and guide the βGal exit. **c** Comparison between the SapB:Gb3-NBD dimer and the SapB crystallographic pair observed in the SapB:GLA binary complex. Arrows indicate the translations on the respective chains to move from the SapB dimer to the pair formation. The relative distances between the Asn21 Cβ (spheres) are shown for reference. **d** Alignments of SapA (blue), SapC (yellow) and SapD (green) crystal structures to the SapB (orange) chain making major contacts with the GLA dimer (light and dark grey). The models are organized by the saposin conformation and regions of major or minor clashes are indicated (white ovals).

bioRxiv preprint doi: https://doi.org/10.1101/2024.04.04.584535; this version posted April 4, 2024. The copyright holder for this preprint (which was not certified by peer review) is the author/funder, who has granted bioRxiv a license to display the preprint in perpetuity. It is made available under aCC-BY 4.0 International license.

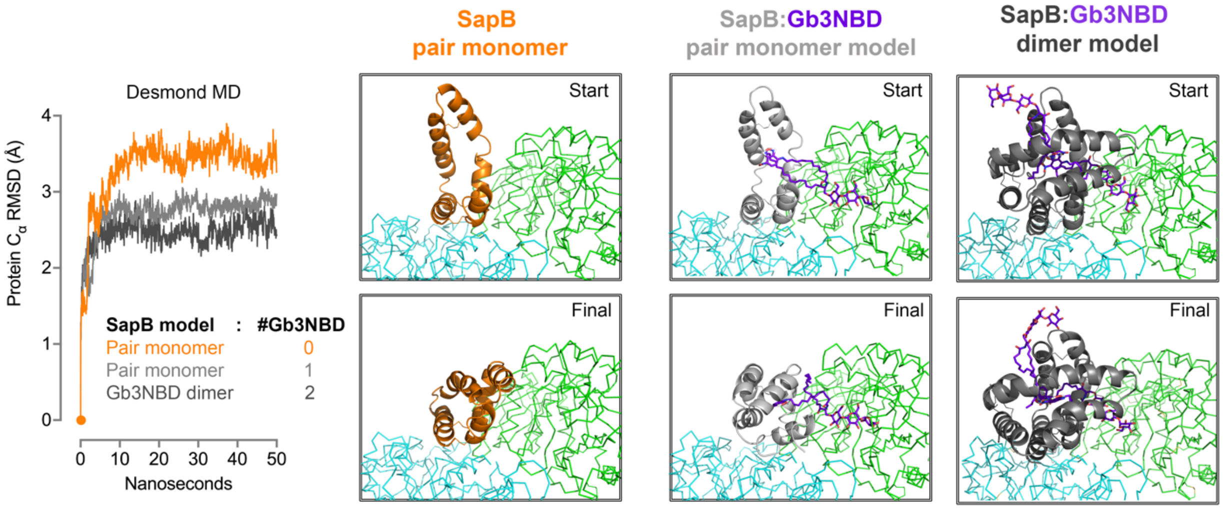

Supplementary Figure 6 | Molecular dynamics on the SapB:GLA binary complex provides models for substrate presentation
Simulations on the asymmetric unit components of the SapB:GLA crystals, containing a SapB monomer and GLA dimer, were conducted in the presence and absence of a single, modeled Gb3-NBD ligand before assessing the compatibility of a SapB dimer. *Left:* The total alpha carbon RMSD of the SapB and GLA chains is graphed by nanosecond after the MD simulation. The starting structure (orange dot) of each simulation is indicated. *Right:* Translations in the SapB chain (cartoon) from the start to the final state after 50 nanoseconds, both in the absence (orange) and presence (light grey) of a single Gb3-NBD molecule (purple stick), are shown. The GLA chain motions are shown in ribbon (green & cyan). A final simulation was rendered of a ligand-loaded SapB:Gb3-NBD dimer (dark grey), aligned to the SapB monomer in the crystal and slightly adjusted to avoid a minor hairpin clash into GLA.
